# Clinical and survival analysis of 36 cases of primary fallopian tube carcinoma

**DOI:** 10.1186/1477-7819-12-311

**Published:** 2014-10-12

**Authors:** Ying Ma, Wei Duan

**Affiliations:** Obstetrical Department, Beijing Obstetrics and Gynecology Hospital, Capital Medical University, 251, Yaojiayuan Street, Chaoyang District, Beijing, 100026 China; Oncology Department, Beijing Obstetrics and Gynecology Hospital, Capital Medical University, 251, Yaojiayuan Street, Chaoyang District, Beijing, 100026 China

**Keywords:** Primary fallopian tube carcinoma, CA125, Risk factors

## Abstract

**Background:**

Primary fallopian tube carcinoma (PFTC) is rarely seen clinically. Herein, we investigate the clinical and pathological characteristics and appropriate therapies for PFTC.

**Methods:**

A total of 36 patients for whom PFTC was pathologically confirmed from January 2001 to July 2011 in Beijing Hospital of Gynecology and Obstetrics, an affiliate of Capital Medical University, were retrospectively analyzed.

**Results:**

A total of 36 cases underwent surgical staging in our hospital: 47.2% were early stage cases , and 52.8% were advanced stage cases. Of the 36 cases, 24 cases were pure adenocarcinoma, 10 cases were mixed, and there was 1 case of undifferentiated carcinoma, 1 case of undifferentiated carcinoma combined with transitional cell carcinoma, 5 cases of moderately differentiated carcinoma, and 29 cases of moderately to poorly differentiated carcinoma. There were no cases of highly differentiated carcinoma. Among the cases examined, 38.9% (14/36) had omentum metastasis, and 19 cases had an elevated CA125 during a preoperative biochemical laboratory test. Approximately 35 cases received postoperative adjuvant chemotherapy. The 3-year and 5-year overall survival rates for the 36 cases were 80.7% and 65.4%, respectively. Single-factor analysis showed that the pathological conditions of residual tumor diameter >1 cm (*P* <0.001), omentum metastasis (*P* = 0.003), ovary metastasis (*P* = 0.004) and elevated preoperative CA125 (*P* = 0.044) were associated with prognosis, whereas pathological surgical staging (*P* = 0.069), retroperitoneal lymph node metastasis (*P* = 0.499), and pathological classification (*P* = 0.183) were not associated with prognosis. Multifactor analysis showed that a residual tumor diameter >1 cm (*P* = 0.019) and omentum metastasis (*P* = 0.015) were associated with prognosis, and were, therefore, the independent risk factors of prognosis.

**Conclusions:**

PFTC is a rare female genital tract malignancy. Most patients are in an advanced stage at diagnosis, which results in a poor prognosis. Complete surgical staging and maximal resection should be recommended.

## Background

Fallopian tube cancer is a rare malignancy, accounting for 0.5% of female genital cancer. It is divided into primary and secondary carcinoma, of which secondary malignancies are more often the results of metastasis of tubal ovarian cancer or endometrial stumbled cancer [1]. Primary fallopian tube carcinoma (PFTC) is rarely seen clinically due to hidden lesions, an absence of recommended screening methods, and relatively limited clinical experience, to the extent that a number of cases have been accidentally detected during other gynecological surgeries [2]. Because the tubal wall has considerable flexibility, even if the tube is filled with fluid or tumor, the tubal wall can still remain quite complete. Intraluminal tubal fluid can be discharged through the uterus-vagina route when fimbria atresia occurs, and this is usually found in early cases. In case of an open fimbrial end, the tumor cells would come into the abdominal cavity where they could easily grow, and this is found mostly in advanced cases, where the prognosis is rather poor.

The etiology of this cancer is unknown. Hormonal, reproductive, and possibly genetic factors thought to increase epithelial ovarian cancer (EOC) risk might also increase PFTC risk [3–5]. High parity has been reported to be protective [6], and a history of pregnancy and the use of oral contraceptives decreases the PFTC risk significantly [7, 8]. It has been reported that there is no statistically significant correlation between PFTC and age, race, weight, education level, pelvic inflammatory disease, infertility, previous hysterectomy, endometriosis, lactose intolerance, or smoking [7, 9–11]. Meng *et al*. [12] found a fivefold higher bilateral occurrence in infertile patients than in fertile patients, and Clayton *et al*. [13] reported a better prognosis in nulliparous women.

In the past, because the incidence of PFTC is fairly low, and because there were no standard diagnostic and staging criteria available, previous publications in this area usually have been in the form of individual case reports. In recent years, with the availability and wide adoption of FIGO staging in our hospital, and the significant enhancement of the accuracy of diagnosis, a comprehensive retrospective analysis has been allowed to help us gain a better understanding of the clinical and pathological characteristics of PFTC.

In the present study, we summarize the clinical information of 36 patients in one institute, and present a thorough analysis of the factors that potentially influenced survival.

## Methods

### Subjects and inclusion criteria

This retrospective study was approved by the ethnic committee of Beijing Obstetrics and Gynecology Hospital, Capital Medical University at 2008; the ethnic committee approved related screening, treatment, data collection and follow-up of these patients. All subjects signed written informed consent form for using their case data.

Participants were recruited from the patients diagnosed with genital malignancies from January 2001 to July 2011 at the Beijing Hospital of Gynecology and Obstetrics, which is affiliated with the Capital Medical University. According to the pathological standards of FIGO, patients with fallopian tube carcinoma metastasis were excluded. A total of 36 patients with pathologically confirmed PFTC were included in the study.

### General demographic data

As shown in Table 1, the mean age at onset was 57.2 years (range: 42 to 72 years). Among these cases, 25 were postmenopausal (69.4%), with a mean period since menopause of 10.8 years (range: 1 to 22 years). There were 9 cases of postmenopausal vaginal bleeding or irregular vaginal bleeding (25.0%), 12 cases of vaginal discharge (33.3%), 9 cases of abdominal distension or abdominal pain (25.0%), 1 case of back pain, and 4 cases (11.1%) were asymptomatic with a pelvic mass being discovered on physical examination. The remaining case underwent uterine-rectal fossa tumor resection under laparoscopy 3 years ago, with poorly differentiated carcinoma being pathologically confirmed. This patient later underwent intraperitoneal chemotherapy with cisplatin once, and upon thorough systemic examination, no primary tumor was found. A year later, a type-B ultrasonic check suggested the presence of a tumor 2 cm in diameter in the right accessories, and right fallopian tube cancer was found after surgery. A total of 28 patients underwent a preoperative cervical ThinPrep cytology test (TCT) examination, and 6 patients underwent curettage.

**Table 1:** **Demographic data of the 36 patients**

Demographic character	Data
Age (years):	Mean: 57.2, Range: 42 to 72
Menopause:	25 postmenopausal (69.4%)
Pathology:	9 postmenopausal vaginal bleeding or irregular vaginal bleeding (25.0%)
	12 vaginal discharge (33.3%)
	9 abdominal distension or abdominal pain (25.0%)
	1 back pain
	4 asymptomatic yet with pelvic mass (11.1%)
	1 poorly differentiated carcinoma
Preoperative screening:	28 preoperative cervical TCT 6 curettage
Surgical method:	1 left oophorectomy and partial omentum resection
	35 uterine double oophorectomy
	31 pelvic an/or aortic lymph node dissection
	35 full or partial omental resection
	20 cytoreductive surgery
Adjuvant therapies:	
Chemotherapy:	1 one-course preoperative neoadjuvant chemotherapy
	35 four- to eight-course postoperative chemotherapy
	31 TP
	4 cisplatin and PC, or cisplatin, PC and PAC
Radiotherapy:	1 radiotherapy after six-course chemotherapy

*Abbreviation*: *TCT* thinpprep cytology test, *TP* taxol and cisplatin, *PC* cisplatin and cyclophosphamide, *PAC* cisplatin, adriamycin and cyclophosphamide.

## Treatments

### Surgical methods

One case underwent left oophorectomy and partial omentum resection due to pelvic tumor adhesions. The same patient, after three courses of chemotherapy, underwent additional surgery and tumor debulking surgery. A total of 35 patients underwent uterine double oophorectomy, and of these, 31 patients simultaneously underwent pelvic and (or) aortic lymph node dissection or sampling. A total of 35 patients underwent full or partial omental resection. And, 20 patients underwent oncocytoreductive surgery. The fallopian tube cancer surgical staging referenced the FIGO (2000) staging criteria.

### Chemotherapy

One patient underwent surgery after one course of neoadjuvant chemotherapy. Another 35 patients underwent 4 to 8 courses of postoperative chemotherapy; of these, 31 patients used taxol and cisplatin (TP) and 4 patients used cisplatin and cyclophosphamide (PC) or cisplatin, cisplatin, adriamycin and cyclophosphamide (PAC).

### Radiotherapy

One patient underwent radiotherapy after six courses of chemotherapy because of a postoperative residual aortic lymph tumor, for which the tumor-absorbed dose (DT) of extracorporeal pelvic irradiation field and extended irradiation field combined was 40Gy. Other patients did not undergo radiotherapy.

### Statistical analysis

SPSS 18.0 software package (Illinois, USA) was used for statistical analyses. The survival rate was calculated using the Kaplan-Meier method. The Kaplan-Meier method and Log-rank test were used in the single-factor analysis of the correlation between the survival rate and pathological staging of the surgery, diameter of an intraoperative residual tumor being more than 1 cm, retroperitoneal lymph node metastasis, ovarian metastasis, pathological classification, omentum metastasis and elevated preoperative CA125. A COX proportional hazards regression model was used in the multivariate analysis of the correlation between the survival rate and an intraoperative residual tumor diameter >1 cm, ovarian metastasis, omentum metastasis, and elevated preoperative CA125. For *P* <0.05, the difference is considered statistically significant.

## Results and discussion

Most clinical characteristics are summarized in Table 2, followed by the respective descriptions.

**Table 2:** **Summary of clinical characteristics**

Clinic indexes	Data
Preoperative screening:	
28 preoperative cervical TCT	1 CIN stage III
	3 with atypical squamous cells
	1 inflammation
	2 cervicitis
6 curettage	1 with necrotic tissues and pelvic mass
	1 uterine clear cell carcinoma
	1 endometrial adenocarcinoma
	2 no abnormal tissues
	1 endometrium not found
Pathology:	
Type:	1 undifferentiated carcinoma
	1 undifferentiated and transitional cell carcinoma
	34 adenocarcinoma
	24 pure adenocarcinoma
	10 mixed type
Complication:	7 complicated with clear cell carcinoma
	2 complicated with endometrial cancer
	1 complicated with transitional cell carcinoma
Differentiation:	5 moderately differentiated
	29 poorly to moderately, or simply poorly, differentiated
	2 undifferentiated
Sites:	33 unilateral fallopian cancer
	3 bilateral fallopian cancer
	13 with involvement in ovary
	4 with uterine transfer
	3 with myometrial invasion
	1 with endometrial involvement
	14 with pelvic metastasis in omentum
	8 with retroperitoneal lymph node metastasis
Surgical Staging:	
Early stage: (47.2%)	4 stage Ia
	5 stage Ic
	2 stage IIa
	1 stage IIb
	5 stage IIc
Advanced Stage: (52.8%)	2 stage IIIb
	17 stage IIIc
Intraoperative residual tumor:	18 with residual tumor
	7 with diameter more than 1 cm
	11 with diameter less than 1 cm
	14 without residual tumor
	4 without records
CA125:	19 had elevated preoperative CA125
	2 stage Ic
	3 stage IIc
	1 stage IIIb
	13 stage IIIc
Follow-up and relapse:	
General:	4 drop-outs
	12 with relapse or metastasis (33.3%)
	10 death
	1 stage Ic
	1 stage IIc
	2 stage IIIb
	6 stage IIIc
	2 live with tumor
Sites of relapse:	1 on vaginal stump
	4 with pelvic relapse
	4 with peritoneal relapse
	3 with significantly elevated CA125 yet no lesions found

*Abbreviation*: *CIN*, cervical intraepithelial neoplasia.

### Preoperative screening

In the results of the cervical TCT examinations that were attended by 28 patients, there was one case suggesting CIN III staging, which was pathologically confirmed by colposcopy. Three cases was found with atypical squamous cells, among which one case was confirmed by colposcopy as having inflammation; the other two cases did not undergo colposcopy, and postoperative pathology suggested cervicitis. Six patients underwent curettage because of vaginal bleeding. One patient underwent curettage twice due to postmenopausal vaginal bleeding, suggesting necrotic tissues, and 10 months after the second curettage, a pelvic mass was found, which was confirmed by laparotomy. One patient underwent curettage and uterine clear cell carcinoma was suggested. For one patient, endometrial adenocarcinoma was suggested. Two patients underwent curettage with no abnormal tissues being found. For one patient, endometrium was not found during curettage.

### Pathology analysis

In the 36 patients who received pathological examinations, there was one patient with undifferentiated carcinoma, one patient with undifferentiated and transitional cell carcinoma, and 34 patients with adenocarcinoma, among whom 24 patients had pure adenocarcinoma and 10 patients had mixed type. Seven patients were complicated with clear cell carcinoma, two patients were complicated with endometrial cancer, and one patient was complicated with transitional cell carcinoma. There were five patients with moderately differentiated carcinoma, 29 patients with poorly to moderately or simply poorly differentiated carcinoma, and 2 patients with undifferentiated carcinoma. There was not a single case with highly differentiated carcinoma. There were 33 patients with unilateral fallopian cancer; 3 patients with bilateral fallopian cancer; 13 patients with involvement of an ovary; 4 patients with uterine transfer, including 3 patients with myometrial invasion and one patient with endometrial involvement; 14 patients with pelvic metastasis in the omentum; and 8 patients with retroperitoneal lymph node metastasis. Ascites occurred in 13 patients, with the volume of ascites ranging from 100 ml to 8,000 ml. There were 19 patients positive for ascites or peritoneal washings.

### Surgical staging and residual tumor

Surgical pathological staging of the 36 patients has referenced the FIGO (2000) standard, with 4 patients as stage Ia, 5 patients as stage Ic, 2 patients as stage IIa, one patient as stage IIb, 5 patients as stage IIc, 2 patients as stage IIIb, and 17 patients as stage IIIc. Early stages account for 47.2% of all patients. For intraoperative residual tumors, there were 18 patients with a residual tumor visible to the naked eyes, with 7 patients having a residual tumor with a diameter greater than 1 cm and 11 patients having a residual tumor with a diameter less than 1 cm. There were 14 patients without residual tumors visible to the naked eyes and 4 patients with no descriptions of residual tumors in their surgical records.

### CA125 examination as tumor marker

The normal reference value of CA125 is greater than 35 kU/L. There were 33 patients who underwent preoperative biochemical laboratory tests, with CA125 ranging from 11.7 to 7,600.0 kU/L. Among these 33 patients, 19 had elevated preoperative CA125, including 2 patients who were stage Ic, 3 patients who were stage IIc, one patient who was stage IIIb and 13 patients who were stage IIIc. For patients in the advanced stages, there were five cases without elevated CA125.

### Adjuvant treatment

One patient with an intraoperative residual tumor less than 2 cm in diameter underwent surgery after one course of neoadjuvant chemotherapy. There were 35 patients who underwent four to eight courses of postoperative chemotherapy; of these patients, 31 used TP, and 4 used cisplatin and cyclophosphamide (PC) or cisplatin, PC and cyclophosphamide (PAC). There were 33 patients assessable for clinical efficacies, with general efficacy of adjuvant treatment reaching 87.9%. One patient underwent radiotherapy after six courses of chemotherapy due to the presence of a postoperative residual aortic lymph tumor, for which the tumor-absorbed dose (DT) of extracorporeal pelvic irradiation field and extended irradiation field combined was 40 Gy. Other patients did not undergo radiotherapy.

### Follow-up and relapse

Follow-up began from the month of the diagnosis and ended in December 2013. The average follow-up period was 51.2 months and ranged from 14 to 125 months. There were four cases of drop-out due to loss of contact, accounting for 11.1% of the patients. During follow-up, there were 12 patients (33.3%) with relapse or metastasis, 10 patients who died of cancer, and 2 patients who have lived with the tumor. The time of relapse or metastasis varied from 1 to 39 months after chemotherapy. Sites of relapse are described as follows: one patient showed relapse on the vaginal stump, four patients showed pelvic relapse, four patients showed peritoneal relapse, and three patients showed relapse with significantly elevated CA125 but without specific lesions being found.

### Survival analysis

**Figure 1:**
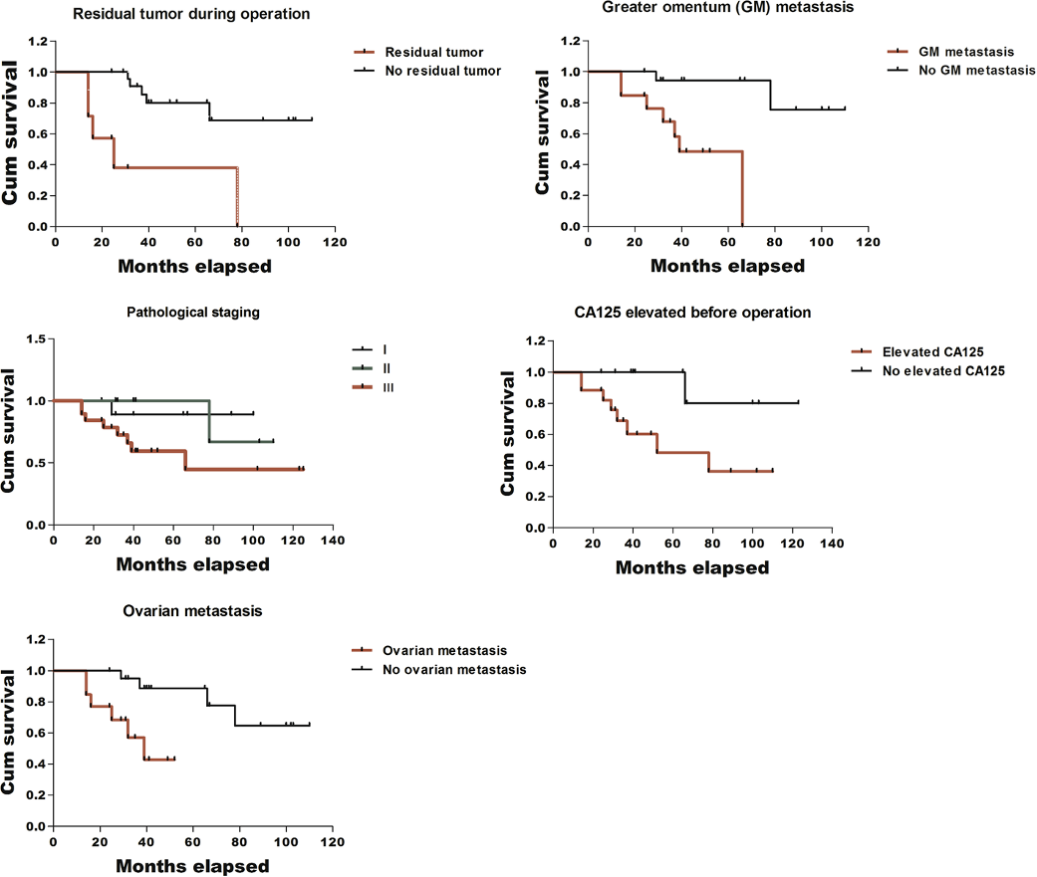
**Five different survival analyses of 36 patients based on different clinical characteristics.**

All the survival analyses were summarized in Figure 1. We did survival analysis based on different clinical characteristics, including with or without residual tumor, with or without greater omentum metastasis, preoperative CA125 content, pathological staging and ovarian metastasis. There were 10 cases of death during the follow-up period, among whom was one patient in the Ic stage, one patient in the IIc stage, two patients in the IIIb stage and 6 patients in the IIIc stage. The early mortality rate (including stage I and II) was 11.8%, and advanced mortality rate (stage III) was 42.1%. The overall survival rates for 3 years and 5 years were 80.7% and 65.4%, respectively. Single-factor analysis showed that pathological conditions of a residual tumor diameter >1 cm (*P* <0.001), omentum metastasis (*P* = 0.003), ovary metastasis (*P* = 0.004) and elevated preoperative CA125 (*P* = 0.044) were associated with prognosis, whereas pathological surgical staging (*P* = 0.069), retroperitoneal lymph node metastasis (*P* = 0.499), and pathological classification (*P* = 0.183) were not associated with prognosis. Multifactor analysis showed that a residual tumor diameter >1 cm (*P* = 0.019) and omentum metastasis (*P* = 0.015) were associated with prognosis, and were therefore the independent risk factors of prognosis.

Fallopian tube cancer is often asymptomatic, with often atypical signs, and the ‘triad’ of vaginal discharge, abdominal pain and pelvic mass. Abnormal vaginal bleeding is also common, with other symptoms including lower abdominal discomfort and abdominal distension. It is reported that PFTC accounts for 0.75 to 1.2% of the malignant tumors of the female reproductive system [14–16]. In our study, results show that the average age of onset is 57.2 years old (ranging from 42 to 72 years old), which is consistent with previous reports of 53 years old [17] and 54.6 years old [18, 19]. Our study shows that 25 patients were postmenopausal (69.4%), which is consistent with reported data [17]. Thus, we may conclude that postmenopausal women are more prone to this disease. There were four patients (11.1%) who were asymptomatic, yet had a pelvic mass that was found during medical examination, suggesting that this disease has an insidious onset. The clinical manifestations of fallopian tube carcinoma are somewhat heterogeneous, and the incidence rate is rather low; thus, the preoperative misdiagnosis rate is high. In this study, 28 patients underwent cervical TCT examination, with only 1 case being identified as CIN grade III, whereas other patients had not shown any abnormal manifestations. Six patients underwent curettage, with only two cases suggesting endometrial cancer. These results suggest that preoperative TCT and curettage can exclude other gynecological malignant tumors, such as cervical cancer or endometrial cancer, but they do not help in the diagnosis of PFTC, which is consistent with the FIGO guidance [20].

Because the oviduct is very close to the ovaries and uterus, it is sometimes difficult to identify primary lesions in patients with advanced carcinoma. In patients with fallopian tube cancer, the ovaries and endometrium can be normal, or they can also have tumor spread to them because of the close lymphatic communication among the three; but, the sizes of the tumor in ovaries and endometrium would be smaller than the tumor in the fallopian tube. The patients in our research were classified according to the WHO standard classification: 5 patients with moderately differentiated carcinoma, 29 patients with moderately to poorly or simply poorly differentiated carcinoma, and 2 patients with undifferentiated carcinoma. There were no patients with highly differentiated carcinoma according to this classification. Previous studies [14–16] reported that up to 80.6% of the patients sampled were of poorly differentiated carcinoma, and patients with highly differentiated carcinoma only account for 2.9% of the sample. Yu *et al*. [21] reported that patients with low differentiation accounted for 72.7% of the patient sample, moderate differentiation 27.3% and no patient with high differentiation. Our study is consistent with previous reports, suggesting that carcinoma that is less differentiated is more commonly seen in PFTC. Rabczynski *et al*. reported that most patients with postoperative mortality were pathologically confirmed as having poorly differentiated PFTC [22]. But our study shows that in the 36 patients, 29 patients (80.6%) had moderately to poorly or simply poorly differentiated carcinoma, accounting for the vast majority of the patient sample. Although statistical analysis showed no correlation between pathological classification and prognosis, this might be because of the small number of poorly differentiated cases. The most common site of extrapelvic metastasis is the omentum, and in our study, there were 14 patients with omentum metastasis. Statistical analysis showed that omentum metastasis and ovarian metastasis are correlated with prognosis, suggesting that the prognosis is rather poor for patients with advanced carcinoma. There were eight patients with retroperitoneal lymph node metastasis, but this was not correlated with prognosis.

The normal reference value of CA125 is greater than 35 kU/L. Previous studies demonstrated that PFTC patients have elevated preoperative CA125 [23]. In our study, 33 patients were preoperatively tested for CA125, with results ranging from 11.7 to 7,600.0 kU/L, and among these were 19 patients (57.6%) who had elevated preoperative CA125. There were five advanced patients who did not show elevated CA125 after medical testing. In our research, statistical analysis showed that an elevated preoperative CA125 is correlated with prognosis, and a decreased CA125 during treatment is correlated with clinical efficacy. There was a significant elevation in CA125 during relapse and progression of disease. Previous research indicates that the serum CA 125 level adequately defines the response to chemotherapy and displays good sensitivity and specificity characteristics during the follow-up of patients with PFTC [23].

Current treatment of fallopian tube cancer is mostly mimicking the treatment of ovarian cancer, with surgery as the major clinical arrangement, and where conservative operation is not a recommended practice [20]. For instance, there were two patients in our study who showed only hydrosalpinx during the surgery, which was pathologically confirmed as indicating a malignant tumor postoperatively. In those patients for whom first surgery is not radical enough, a second surgical staging or cytoreductive surgery should be considered. Multivariate analysis showed that the pathological conditions of an intraoperative residual tumor diameter >1 cm (*P* = 0.019) and omentum metastasis (*P* = 0.015) are correlated with prognosis, and were the independent risk factors of prognosis. The FIGO guidance has suggested that the postoperative procedures of PFTC are basically of the same principle with those of the ovarian cancer (That is, patients at stage I who were pathologically highly differentiated do not necessarily have to go through adjuvant chemotherapy) [20]. But clinically, patients of this kind are very rare; thus, the majority of patients have to settle on a plan of paclitaxel combined with platinum. Our study showed that there were 33 patients assessable for clinical efficacy after postoperative chemotherapy, suggesting a general clinical efficacy for chemotherapy of 87.9%. Many patients still have significantly elevated CA125 after cytoreductive surgery, which later drops back down to normal after chemotherapy and remains at the normal level if no relapse or metastasis occurs. Thus, the CA125 level during the postoperative follow-up period is of great significance to the prediction of relapse and metastasis.

Yu *et al*. [21] reported overall 5-year survival rates as 56.3% with a total of 64 patients involved. Other authors have reported that the 3-year and 5-year survival rates of PFTC patients were 87.3% and 65.2%, respectively [18]. Previous studies reported that the 5-year survival rates for PFTC patients of stage I, II, III and IV were 66.7%, 50.0%, 36.0% and 0, respectively. Our study reports that the overall survival rates of PFTC patients for 3-year and 5-year length are 80.7% and 65.4%. In our research, single factor analysis showed that the pathological condition of intraoperative residual tumor diameter >1 cm was correlated with prognosis, which is consistent with previous research [19].

## Conclusions

Although single-factor analysis showed that surgical pathological staging was not correlated with prognosis, the mortality rate of advanced cases (stage III) (42.1%) was significantly higher than early cases (11.8%). Our research also showed that omentum metastasis and ovarian metastasis were correlated with prognosis, suggesting that the survival potential of advanced cases seems gloomy. This is evidenced in the report by Tulunay [24]. Multivariate analysis showed that the pathological conditions of an intraoperative residual tumor diameter >1 cm (*P* = 0.019) and omentum metastasis (*P* = 0.015) were correlated with prognosis, and that these were independent risk factors of prognosis. Yu *et al*. [21] suggested that the 3-year and 5-year survival rates of those who had received surgical staging were higher than for those who had not received surgical staging. The earlier the surgery, the more radical the results can be. Thus, early diagnosis and early treatment are the key factors to improving prognosis.

To summarize, the incidence of PFTC is rather low, and its clinical manifestations are rather heterogeneous; thus, it is difficult to confirm pathologically before surgery. With the wide adoption of FIGO (2000) staging, the clinical understanding of PFTC becomes clearer and its treatments are increasingly standardized. Whether or not one should carry out a radical cytoreductive surgery, which includes the excision of all primary tumors and metastases, is of critical importance. And, postoperative chemotherapy is an important adjuvant treatment. With these practices standardized, the survival potential of PFTC can be greatly enhanced.

## Abbreviations

CIN: cervical intraepithelial neoplasia
DT: tumor-absorbed dose
EOC: epithelial ovarian cancer
PAC: cisplatin, adriamycin and cyclophosphamide
PC: cisplatin and cyclophosphamide
PFTC: primary fallopian tube carcinoma
TCT: ThinPrep cytology test
TP: taxol and cisplatin.
